# Sucrosomial^®^ Iron Supplementation in Mice: Effects on Blood Parameters, Hepcidin, and Inflammation

**DOI:** 10.3390/nu10101349

**Published:** 2018-09-21

**Authors:** Michela Asperti, Magdalena Gryzik, Elisa Brilli, Annalisa Castagna, Michela Corbella, Rossella Gottardo, Domenico Girelli, Germano Tarantino, Paolo Arosio, Maura Poli

**Affiliations:** 1 Department of Molecular and Translational Medicine, University of Brescia, Viale Europa 11, 25123 Brescia, Italy; michela.asperti@unibs.it (M.A.); m.gryzik@unibs.it (M.G.); maura.poli@unibs.it (M.P.); 2 Pharmanutra S.p.a. 56122 Pisa, Italy; e.brilli@pharmanutra.it (E.B.); g.tarantino@pharmanutra.it (G.T.); 3 Department of Medicine, University of Verona, 37134 Verona, Italy; annalisa.castagna@univr.it (A.C.); michela.corbella@univr.it (M.C.); domenico.girelli@univr.it (D.G.); 4 Department of Diagnostics and Public Health, University of Verona, 37134 Verona, Italy; rossella.gottardo@univr.it

**Keywords:** iron absorption, Sucrosomial^®^ Iron, iron deficient anemia, oral iron, mouse models

## Abstract

Sucrosomial^®^ Iron is a recently developed formulation to treat iron deficiency based on ferric pyrophosphate covered by a matrix of phospholipids plus sucrose esters of fatty acids. Previous data indicated that Sucrosomial^®^ Iron is efficiently absorbed by iron-deficient subjects, even at low dosage, and without side effects. Its structural properties may suggest that it is absorbed by an intestinal pathway which is different to the one used by ionic iron. Although, studies *in vitro* showed that Sucrosomial^®^ Iron is readily absorbed, no animal models have been established to study this important aspect. To this aim, we induced iron deficient anemia in mice by feeding them with a low-iron diet, and then we treated them with either Sucrosomial^®^ Iron or sulfate iron by gavage for up to two weeks. Both iron formulations corrected anemia and restored iron stores in a two-week period, but with different kinetics. Ferrous Sulfate was more efficient during the first week and Sucrosomial^®^ Iron in the second week. Of note, when given at the same concentrations, Ferrous Sulfate induced the expression of hepcidin and four different inflammatory markers (Socs3, Saa1, IL6 and CRP), while Sucrosomial^®^ Iron did not. We conclude that anemic mice are interesting models to study the absorption of oral iron, and that Sucrosomial^®^ Iron is to be preferred over Ferrous Sulfate because of similar absorption but without inducing an inflammatory response.

## 1. Introduction

Iron deficiency is one of the most common nutritional disorders in the world, and leads to mild or severe anemia and serious consequences in different organs with dramatic effects on life [1]. Frontline treatment of iron deficiency anemia generally consists of oral formulations based on ferrous iron, which is readily absorbed, but that often cause gastrointestinal side effects, such as gastric irritation, nausea, and constipation, that reduce compliance and treatment efficacy [2,3]. Such manifestations are supposed to be due to the instability of ferrous iron that readily oxidizes to ferric iron in the presence of oxygen, with the production of reactive oxygen species (ROS), that cause direct toxicity on the intestinal mucosa [4]. Ferric formulations are more stable with lower side effects but are generally poorly absorbed because of the insolubility of ferric iron at neutral pH values [5]. In addition, since only 10–20% of oral iron is absorbed, most of it remains available to gut microbiota with a consequent alteration of its composition that can lead to intestinal inflammation [6,7]. To be absorbed, ferric iron has to be reduced to Fe(II) by the ferric reductase Duodenal Cytochrome b (Dcytb) of duodenal enterocytes, then it passes into the cell cytoplasm via the Divalent Metal Transporter 1 (DMT1) and it is eventually made available to the circulating transferrin after exit via the iron exporter ferroportin expressed at the basolateral side, and re-oxidation to ferric iron by hephaestin [8]. Iron absorption is tightly regulated by feedback mechanisms that include the expression of liver hepcidin and duodenal ferritin, both acting to retain iron inside the enterocytes [5]. Local and systemic inflammation induce a similar response to reduce iron absorption by stimulating hepcidin and ferritin expression [9] as it occurs in the anemia of chronic disease (ACD) or anemia of inflammation (AI) [10]. To bypass the problem of reduced iron absorption, intravenous treatments with ferric iron formulations can be used, but they are costly and not devoid of side effects. Novel oral iron formulations are actively studied to improve the absorption, thus reducing dosage and side effects. Among them, one of the most promising is Sucrosomial^®^ Iron (SI) [3], a preparation of ferric pyrophosphate covered by a phospholipids plus sucrose esters of fatty acids matrix. In experiments using cultured CaCo2 epithelial cells, SI has been shown to be readily absorbed, possibly with a mechanism not involving the DMT1 [11]. Noteworthy, oral SI has been reported effective for treating iron deficiency anemia in patients with conditions classically associated with impaired iron absorption like celiac disease [12], bariatric surgery [13], myelodysplastic syndromes [14], cancer [15,16], or chronic kidney disease [17]. Moreover, recently, Capra and collaborators published a case study of a patient with iron refractory iron deficient anemia (IRIDA), reporting a gradual increase in the patient’s hemoglobin level which remained stable during one year follow-up [18]. Overall, SI was also comparable with intravenous iron gluconate in some clinical settings [16,17]. Thus, SI represents promising tool to treat absolute or functional iron deficiency, but its absorption mechanism remains to be clarified. To this end, we comparatively assessed the efficacy of SI and Ferrous Sulfate (FS), administered via gavage, on improving hemoglobin (Hb) levels and iron status, as well as on hepcidin and inflammatory marker response, in a model of iron deficiency anemia in mice. To this aim, animal models are important, though, surprisingly, mouse models for the study of iron supplement absorption have not been described so far.

## 2. Materials and Methods

### 2.1. Reagents

For the *in vitro* and *in vivo* experiments, FS (Ferrous Sulfate), SI (Sucrosomial^®^ Iron, patent n°PCT/IB2013/001659 owned by Alesco s.r.l, Italy) and vehicle (same composition of Sucrosomial^®^ Iron but without pyrophosphate iron), were provided from Alesco Srl (Pisa, Italy). Ferric Ammonium Citrate (FAC) was from Sigma-Aldrich and saline (0.9% NaCl) was from Baxter Spa (Rome, Italy).

### 2.2. Cells

The human hepatoma cell line, HepG2 (American Type Culture Collection, ATCC, Manassas, VA, USA), and mouse Balb/c monocyte macrophage, J774 (ATCC), were cultured in minimum essential medium (MEM, Gibco, Life technologies, Monza, Italy), and Dulbecco’s Modified Eagle’s Medium respectively, containing 10% endotoxin-free fetal bovine serum (FBS) (Sigma-Aldrich), 0.04 mg/mL gentamicin (Gibco), and 2 mM l-glutamine (Gibco) at 37 °C under an atmosphere of 5% CO_2_/95% air. 

### 2.3. Cell Treatments

HepG2 and J774 cells were seeded in 12-well plates (1.5 × 10^5^ cells/well). After 24 h, the diluted iron formulations or vehicle were added to culture medium and cells incubated for a further 16 h. To simulate gastric digestion, the iron samples were treated as reported elsewhere [11], and the final suspension was immediately used to treat the cells or kept frozen at −20 °C. After the treatment the cells were harvested and samples of the homogenates separated on nondenaturing PAGE for analysis of ferritin content using Western blotting and of ferritin iron by Prussian Blue staining. All experiments were performed in triplicate.

### 2.4. Mice Treatments

C57BL/J6 mice (Harlan Laboratories, Bresso, Italy) were housed in an Association for Assessment and Accreditation of Laboratory Animal Care approved facility at the University of Brescia, Italy, following standards and procedures approved by the Institution Animal Care and Use Committee for ethical use of animals in experiments. 

Healthy mice: Female C57BL/J6 mice seven-week old, maintained on an iron balance diet containing 200 mg/kg of carbonyl-iron (Scientific Animal Food & Engineering, SAFE, Augy, France) were treated daily by gavage with about 150 µL of saline, vehicle (same composition of SI but without pyrophosphate iron), and 1 mg/kg of SI or FS every day for two weeks. All the treatments were carried out at about the same time in the morning. The mice were weighed every morning before treatment and the volume of SI and FS administered was adjusted to the weight of each mouse but never higher than 150 µL. A prolonged treatment of four weeks was attempted to verify if it may induce iron accumulation. We used five mice per experimental group. 

Anemic mice: To induce iron deficiency anemia four-week old C57BL/J6 female mice were kept on a low-iron diet containing 5–5.9 mg/kg Carbonyl Iron (Cod. U8958P Version 0176, from SAFE) for 6 to 8 weeks. Hb and Ht was monitored weekly using Hemo_Vet Instrument (InfraTec, Dresden, Germany) by collecting a single drop of blood. The mice showed baseline Hb levels of 15.5–17 g/dL, and the iron treatment started after Hb fell below 12.5 g/dL. It consisted in daily subministration by gavage of saline, vehicle (same composition of SI but without pyrophosphate iron), SI or FS for two weeks. All the treatments were carried out at about the same time in the morning. The mice were weighed every morning before treatment and the amount of SI and FS administered was adjusted on the weight of each mouse but never higher than 150 µL.

Mouse serum hepcidin was quantified using a validated mass-spectrometry based assay [19,20], recently updated [21,22]. Serum iron and transferrin saturation were determined spectrophotometrically with a commercial kit, according to the manufacturer’s instruction (Randox Laboratories).

### 2.5. Immunoblot Analysis

Liver and spleen tissues or cells extracts were prepared using RIPA buffer (150 mM NaCl, 1% NP-40, 0.5% sodium deoxycholate, 0.1% sodium dodecylsulfate, 50 mM Tris pH 8.0, 50 mM dithiothreitol, 0.01 mg/mL leupeptin, and Protease Inhibitor Cocktail (Roche) or using a lysis buffer (200 mM Tris-HCl pH 8.0, 100 mM NaCl, 1 mM EDTA, 0.5% NP-40, 10% glycerol, 1 mM sodium fluoride, 1 mM sodium orthovanadate, and Complete Protease Inhibitor Cocktail (Sigma-Aldrich, Milan, Italy). Protein was quantified by BCA assay (ThermoScientific-Pierce, Spinea, Italy). Samples (40–50 µg) were separated on 10–14% SDS-PAGE and transferred to Hybond-P Membrane (GE, Milan, Italy). The primary antibodies used for immunoblotting were: anti-l-ferritin (SIGMA #F5012), anti-GAPDH (ORIGENE #TA802519), and anti-actin (ORIGENE #811000). After incubation with horseradish peroxidase-conjugated secondary antibodies, membranes were developed with SuperSignal West Pico Chemiluminescent Substrate (ThermoScientific-Pierce) and visualized with Lycor Odyssey instrument. Densitometric analysis was performed using ImageJ software (NIH, Bethesda, MD, USA) normalized against actin (for HepG2, J774 and Spleen) and GAPDH (for liver samples).

### 2.6. Quantitative qRT-PCR

Total RNA was isolated from liver tissues using TRIzol Reagent (Ambion), according to the manufacturer’s instruction. cDNA was generated by Reverse transcription, using 1 µg RNA and Improm Reverse Transcriptase (Promega, Milan, Italy) in 20 µL. Samples were analyzed by quantitative reverse transcription polymerase chain reaction (qRT-PCR), using PowerUp SYBR Green Master Mix (Life Technologies) according to the manufacturer’s instructions. All data are normalized to expression of HPRT1 and expressed as relative quantification (method of 2^−ΔΔ*C*t^). The primers used are: 

Mm-Hprt1 For 5-CTGGTTAAGCAGTACAGCCCCAA-3, and Rev 5-CAGGAGGTCCTTTTCACCAGC-3; 

Mm–Hep For 5-AAGCAGGGCAGACATTGCGAT-3 and Rev 5-CAGGATGTGGCTCTAGGCTATGT-3; 

Mm-Socs3 For 5-TTAAATGCCCTCTGTCCCAGG-3 and Rev 5-TGTTTGGCTCCTTGTGTGCC-3; 

Mm-CRP For 5-GCTACTCTGGTGCCTTCTGATCA-3 and Rev 5-GGCTTCTTTGACTCTGCTTCCA-3. 

Mm-IL6 For 5-CTCTGCAAGAGACTTCCATCCAGT-3 and Rev 5-CGTGGTTGTCACCAGCATCA-3;

Mm-Saa1 For 5-AGAGGACATGAGGACACCAT-3 and Rev 5-CAGGAGGTCTGTAGTAATTGG-3.

### 2.7. Iron Quantification

Tissue iron content were determined spectrophotometrically as previously described [23] with minor modifications. Briefly, 50 mg of tissue was incubated for 18 h at 65 °C in 3 M HCl and 0.6 M trichloroacetic acid. After centrifugation, a 10 µL sample of clarified acid extract was added to 240 µL of working chromogen reagent containing 1 vol of 0.1% bathophenanthroline sulfonate/1% thioglycolic acid solution, 5 vol of water, and 5 vol of saturated sodium acetate in a 96 well/plate. The solutions were then incubated for 30 min at room temperature and the absorbance measured at 535 nm in a plate reader. A standard curve was prepared with a precalibrated solution of FeCl_3_ (Sigma-Aldrich). 

### 2.8. Ferritin Iron Determination

Cell lysates, liver and spleen homogenates were heated at 70 °C for 10 min to enrich ferritins. Samples (equivalent to 50 µg of preheated protein) were loaded on 8% non-denaturing PAGE and run for 3 h at 160 V. The gels were washed with water and incubated in 2% ferrocyanide (Sigma-Aldrich) and 2% HCl for 1 h. To enhance the signal, the gels were incubated in 0.025% 3,3′-diaminobenzidine (Sigma-Aldrich) and 0.05% H_2_O_2_ in 20 mM Tris HCl, pH 7.4 for 15–30 min. The reaction was blocked by washing with water.

### 2.9. ELISA for Mouse l-Ferritin

l-ferritin was also quantified by an in-house ELISA assay. The rabbit anti-mouse Ft-l antiserum was absorbed on the 96-well microplate by adding 100 µL of 10 µg/mL in 50 mM sodium carbonate, pH 9.6 at 4 °C for 18 h or at 37 °C for 2 h. After three washes with 200 µL of phosphate-buffered saline (PBS) with 0.1% Tween (PBST), the wells were overcoated by adding 100 µL of 3% bovine serum albumin (BSA) in PBS for 1 h at 37 °C. After washing, 100 µL of 10–20 µg protein extracts from liver and spleen in PBST were added to the wells and incubated for 2 h at 37 °C. After wash, 100 µL of HRP-conjugated anti-Ft-l antibody at 1:500 dilution was added and incubated for 1 h at 37 °C. HRP activity was detected using 1 mg/mL tetramethylbenzene (TMB) in dimethyl sulfoxide (DMSO) diluted 1:10 with phosphate-citrate buffer, pH 5 with added fresh hydrogen peroxide to final concentration 0.006% and the absorbance read at 620 nm by MultiskanEx plate reader (Thermo). The reaction was stopped by adding 1 N sulphuric acid and the absorbance was measured at 405 nm. The assay was calibrated using various dilutions of purified recombinant mouse l-ferritin.

### 2.10. Statistics

Data are shown as mean ± standard deviation (±SD) or standard error of mean (±S.E.M.) as indicated in the figures. Generally, expression levels were scaled to control values and expressed as fold change or percentage. Comparison of values between untreated or treated cells as well as comparison between saline or vehicle and SI and FS treated mice were performed by unpaired, two-tailed Student *t* test or one-way ANOVA. Multiple comparisons were corrected by Tukey’s test (GraphPad Prism6, GraphPad Software, Inc., La Jolla, CA, USA). Differences were defined as significant for *p* values < 0.05.

## 3. Results

### 3.1. Cell Treatments with the Digested Iron Preparations

**Figure 1:**
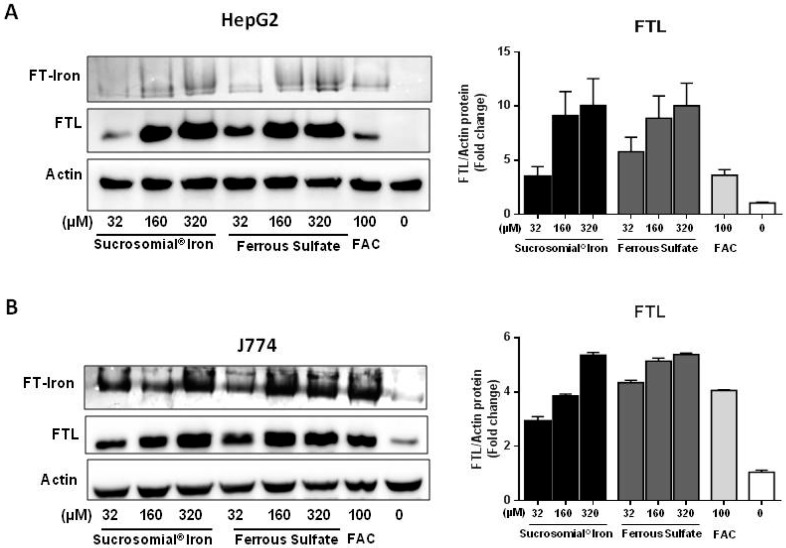
Sucrosomial^®^ Iron and Ferrous Sulfate are absorbed by HepG2 and J774 cells. HepG2 (**A**) and J774 (**B**) cells were treated with increasing concentrations of Sucrosomial^®^ Iron, Ferrous Sulfate and with 100 μM ferric ammonium citrate (FAC) for 16 h, and then the homogenates run on denaturing PAGE for ferritin (FTL) and nondenaturing PAGE for ferritin iron (FT-Iron) evaluation. For ferritin iron content (FT-iron) a representative image of Prussian blue stain plus DAB enhancement is shown for HepG2 (**A** top) and for J774 (**B** top). The expression of l-ferritin (FTL) was analyzed in Western blotting in HepG2 (**A** bottom) and J774 (**B** bottom) and the densitometry was performed using ImageJ. The values (*N* = 3) were normalized to actin.

Sucrosomial^®^ Iron (SI) and Ferrous Sulfate (FS) were digested in a gastric environment before being added to hepatoma (HepG2) and macrophagic (J774) cell lines with a procedure that was shown not to affect the structure of the Sucrosomial^®^ Iron particles [11]. Different iron concentrations (32–160–320 µM) of SI or FS were added to the cells and incubated for 16 h. Ferric ammonium citrate (FAC), at the concentration of 100 µM normally used for cellular iron supplementation, was used as a reference [24] and as negative control vehicle was used, which consisted in the digested preparation of SI without iron. The cells were harvested and analyzed for l-ferritin and for ferritin-iron content (Figure 1A,B). In HepG2, the induction of l-ferritin and of ferritin-iron of about 10 fold over basal for both SI and FS and about two-fold stronger than that caused by 100 µM FAC (Figure 1A). In J774 cells, the induction of l-ferritin and ferritin-iron by SI and FS was about 6 fold over the basal and comparable to that of the 100 µM FAC. (Figure 1B). The results show that the two iron formulations are efficiently absorbed by the hepatocytic and macrophagic cell lines, as it occurred in CaCo2 cells [11].

### 3.2. Oral Iron Treatments of Mice with Normal Iron Status

To evaluate how SI and FS iron are absorbed *in vivo* in animal models, we first treated seven-week old C57BL6/J mice by daily gavage with ~150 µL of saline or vehicle, SI or FS at a dose of 1 mg/kg for two and four weeks (five mice per group). At the end of the treatment, the mice were sacrificed and blood, liver and spleen collected for the assessment of iron parameters. Compared to saline or vehicle, no changes in serum iron (Figures S1A and S2A) or transferrin saturation (TSat) (Figures S1B and S2B) were observed after two-week treatment with either iron formulations. Similarly, the liver or spleen iron content (Figures S1C,D and S2C,D), l-ferritin protein and ferritin-iron (Figure S3A,B) were not significantly modified by the two-week iron supplementation, and were similar to that observed in saline- or vehicle-treated groups. Then we extended the treatment for another two weeks, but even after this period the indices of iron status did not indicate iron accumulation. Also the values of Hb and Ht were unchanged during the treatment (Table S1). We concluded that healthy mice do not absorb the iron administered by gavage, irrespective of the type of formulation. 

### 3.3. Oral Iron Treatments of Mice with Iron Deficiency Anemia

**Figure 2:**
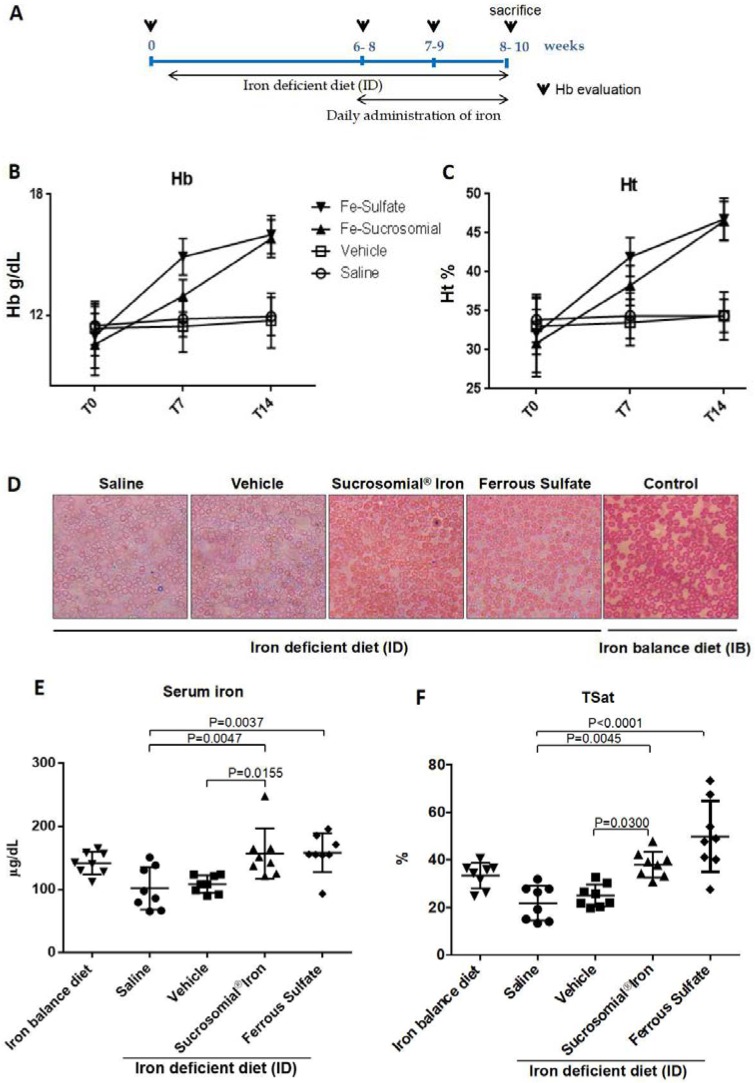
Sucrosomial^®^ Iron and Ferrous Sulfate treatment of anemic mice rescued the anemia. (**A**) Scheme of the treatment protocol: healthy mice were maintained for 6–8 weeks in iron free-diet to reach hemoglobin level <12 g/dL, then in presence of iron free diet they were treated daily with Saline, Vehicle, Sucrosomial^®^ Iron and Ferrous Sulfate (1 mg/kg) by gavage for 2 weeks. (**B**) Hemoglobin and (**C**) hematocrit level in mice after the treatments; (**D**) Representative image of peripheral blood smear (May–Grünwald staining) of treated mice (Saline, Vehicle, Sucrosomial^®^ Iron, Ferrous Sulfate) in presence of iron-free diet (ID) compared to healthy mice (on the right, control mouse) maintained in iron-balanced diet (IB). (**E**) Serum iron content. (**F**) Transferrin saturation (TSat) content in the four treated groups. In E and F Saline (circles), Vehicle (squares), Sucrosomial^®^ Iron (triangles), Ferrous Sulfate (diamonds) and iron balance group (inverted triangles). *p* values were obtained by one-way ANOVA comparing Sucrosomial^®^ Iron and ferrous sulfate versus saline and vehicle.

To verify oral iron absorption under conditions of iron deficiency, mice were maintained in low-iron diet until their hemoglobin levels dropped from the initial value of 15.5–17 g/dL to below 12.5 g/dL, a process that took up to 6–8 weeks. Once anemic the mice were then subjected to daily oral gavage with ~150 µL saline, vehicle, SI (1 mg/kg) and FS (1 mg/kg) for two weeks and maintained on the low-iron diet. A scheme of the treatment is in Figure 2A, which shows that hemoglobin level was evaluated before the low-iron diet (0), after 6–8 weeks when the mice became anemic (6–8), and then in the following two weeks of iron treatment (8–10). Then the mice were sacrificed and the iron parameters analyzed in serum, liver and spleen. As expected, hemoglobin (Hb) and hematocrit (Ht) did not change significantly in the saline- and vehicle-treated groups, while increased significantly to reach normal levels in the SI- and FS-treated groups. The Hb increased similarly with both the formulations at the end of the two weeks but FS caused a faster increase of Hb in the first week (mean Hb 14.3 g/dL) compared to SI (mean Hb 13.0 g/dL) that showed a major increase in the second week (Figure 2B) as visible from the slope of the lines. A similar trend was observed for Ht (Figure 2C). The single values of Hb and Ht per mouse are summarized in Table S2. 

Blood smears stained with May–Grünwald showed that the healthy control mice had many well-stained erythrocytes, while the anemic mice treated with saline and vehicle displayed cells with a central pallor and less intense staining. After the treatment with SI and FS, the erythrocytes appeared similar to those of the healthy control with stronger staining even at the cell center (Figure 2D). This confirmed that saline- and vehicle-treated mice remained anemic, whereas in SI- and FS-treated mice recovered from anemia.

**Figure 3:**
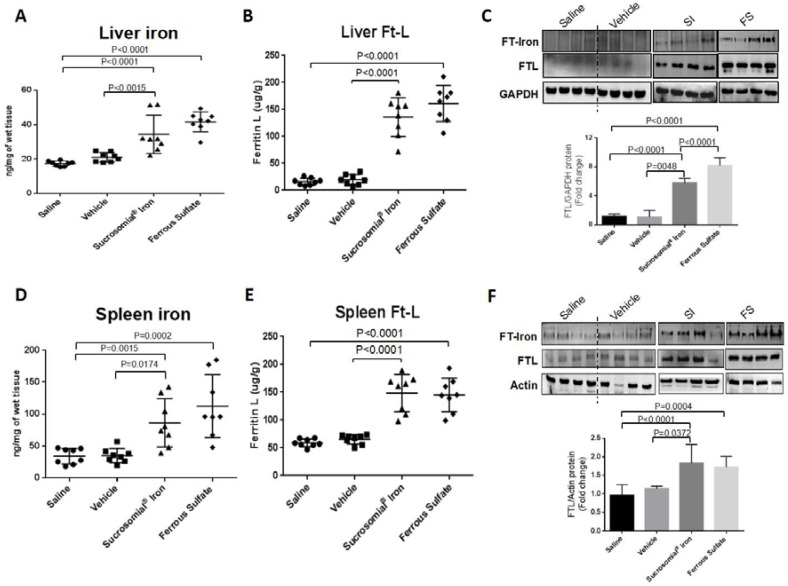
Anemic mice treated with Sucrosomial^®^ Iron and Ferrous Sulfate showed increased body iron stores. (**A**) Liver and (**D**) spleen iron contents were quantified in all groups of mice treated with Saline, Vehicle, Sucrosomial^®^ Iron and Ferrous Fulfate (1 mg/kg) by gavage for two weeks. (**B**) and (**E**) ELISA assay for the quantification of l-ferritin in liver and spleen. In (**A**), (**B**), (**D**) and (**E**) Saline (circles), Vehicle (squares), Sucrosomial^®^ Iron (triangles) and Ferrous Sulfate (diamonds).; (**C**) and (**F**) Representative image of Prussian blue stain plus DAB enhancement of ferritin-iron (FT-Iron) (top) and representative image of Western blotting for l-ferritin in liver and spleen of treated mice. The densitometry was performed using ImageJ. The values (*N* = 3) were normalized to GAPDH or actin for liver and spleen respectively. *p* values were obtained by one-way ANOVA.

**Figure 4:**
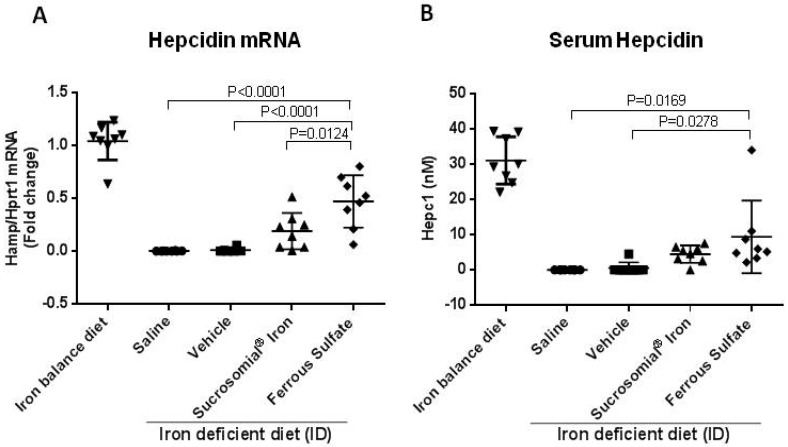
Treatment effect on liver hepcidin mRNA and serum hepcidin. (**A**) qPCR for hepcidin normalized for HPRT1; and (**B**) serum hepcidin by mass spectrometry. Analysis by qPCR were performed on samples derived from liver of healthy untreated mice on an iron balanced diet (IB) and mice on iron deficient diet (ID) treated with Saline, Vehicle, Sucrosomial^®^ Iron and Ferrous Sulfate (1 mg/kg) by gavage for two weeks. In (**A**) and (**B**) Saline (circles), Vehicle (squares), Sucrosomial^®^ Iron (triangles), Ferrous Sulfate (diamonds) and iron balance group (inverted triangles). *p* values were obtained by one-way ANOVA.

**Figure 5:**
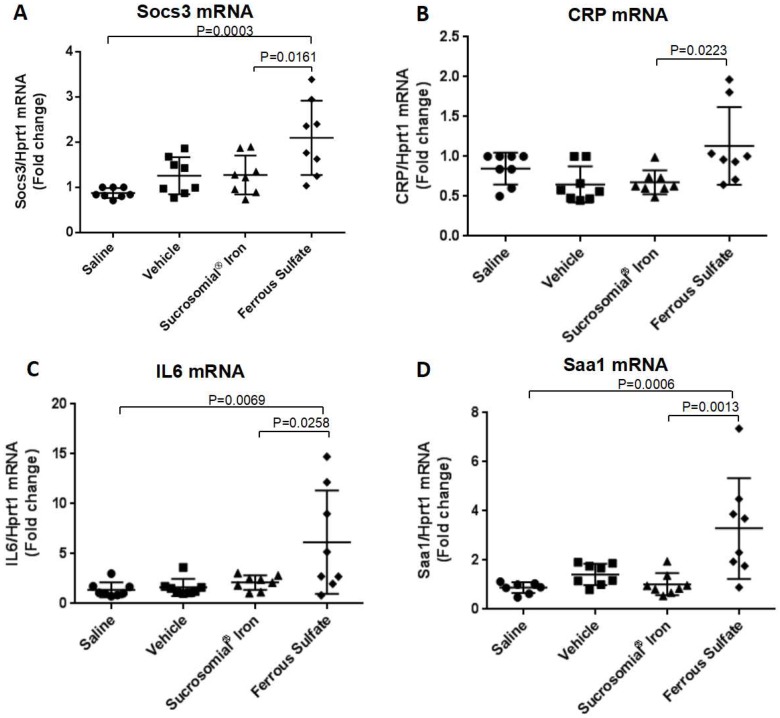
Effects of oral iron supplementation on the inflammatory markers Socs3 CRP, IL6 and Saa1 mRNAs. (**A**) qPCR for Socs3, (**B**) CRP, (**C**) IL6 and (**D**) Saa1 normalized for Hprt1 were performed on samples derived from liver of mice treated with Saline, Vehicle, Sucrosomial^®^ Iron and Ferrous Sulfate (1 mg/kg) by gavage for two weeks. In (**A**), (**B**), (**C**) and (**D**) Saline (circles), Vehicle (squares), Sucrosomial^®^ Iron (triangles), Ferrous Sulfate (diamonds) and iron balance group (inverted triangles). *p* values were obtained by one-way ANOVA.

At the end of the two-week treatment serum iron levels and TSat were low in the saline- or vehicle-treated mice and significantly increased after oral iron supplementation, with a trend to higher values of TSat in the FS-treated group that reached values similar to those of the mice maintained on an iron-balance diet (Figure 2E,F). Similarly, liver and spleen iron concentrations increased after oral iron treatment (Figure 3A,D), and they were accompanied by evident increases of ferritin protein and ferritin-iron levels (Figure 3B,C,E,F). Of interest, the two formulations had different effects on liver hepcidin mRNA and circulating hepcidin levels. The SI-treated mice showed a minor non-significant increase in liver hepcidin mRNA and serum hepcidin levels, but they increased significantly in the FS-treated mice, compared to saline- and vehicle-treated groups (Figure 4A,B). Since liver hepcidin expression is stimulated by inflammation, besides iron, we analyzed the liver expression of the transcripts of four inflammatory markers, Socs3, CRP, Saa1 and IL6. We found that all of them were significantly increased in FS-treated mice but not in SI-treated mice (Figure 5A–D). This suggested that the hepcidin up-regulation is an inflammatory response caused by FS treatment, although in the mice it was used at a dosage lower than that normally used in human. 

## 4. Discussion

Oral administration of Ferrous Sulfate and Sucrosomial^®^ Iron represents the standard front-line treatment and a relatively novel alternative formulation for the oral replacement of human iron deficiency [3]. We compared the two formulations initially in cultured hepatoma and macrophage cell lines and we found that they were taken up by cells with similar efficiency, as indicated by a similar level of induction of l-ferritin protein (FTL) and of ferritin-iron, which are highly sensitive markers of cellular iron status. This is in agreement with recent data showing that SI is efficiently taken up by CaCO_2_ cell lines [11], and stimulated further study on its iron-replacement activity *in vivo*. The first attempt of administration of SI or FS by oral gavage to healthy mice was unsuccessful, since the largely used ferrous sulfate did not induce an evident increase of any of the iron indices we analyzed, even after four weeks of daily administration of 1 mg/kg iron. Also SI had no apparent effect. We concluded that healthy mice do not absorb the iron administered by gavage. Thus we switched to the mice made anemic after being on an iron-free diet for several weeks until their Hb level dropped below 12.5 g/dL. This was not an easy procedure because animal responses to the low-iron diet were very different, few of them became anemic readily, within two weeks, while most took six–eight weeks. We used only the latter ones, females, about 10-week old and of 22–25 g. We found that these anemic mice responded to iron supplementation, thus we used them to compare the efficacy of the two oral iron formulations. The iron supplementation started when mice had an Hb level below 12.5 g/dL, and the major study endpoint was the increase in Hb level. Neither Saline nor Vehicle (negative control treatments) had evident effects on Hb levels or on iron status, while SI and FS treatment did. After two weeks of daily administration of the two formulations, anemia was fully rescued in a rather similar way. We observed slightly higher Hb values in SI-treated mice as compared to FS-treated animals, but this difference was not statistically significant. Both iron preparations restored serum iron levels and replenished iron stores to a similar extent. On the other hand, serial measurements of Hb and Ht during the two-week follow-up indicated different kinetics of the two formulations, with FS apparently having higher effect in the first week with a slower or no increase in the second week and SI having a slower increase in the first week and higher in the second week (Figure 2B and Table S2). The most intriguing difference between the two formulations became apparent in the study of inflammatory indices. While FS treatment led to an increase of CRP, SOCS3, Saa1 and IL6 liver transcript levels, such an effect was not observed in SI-treated mice. It is well-known that ferrous ions can be readily oxidized in the presence of oxygen, with ensuing production of reactive oxygen species (ROS), which in turn can induce an inflammatory response both in the gut and at a systemic level [4]. On the other hand, SI is stable under aerobic conditions and the iron particles are protected by the sucrosomial matrix. This could explain the absence of overt inflammation in SI-treated mice. Such a different behavior could be relevant since inflammation is known to upregulate hepcidin [25], which in turn reduces iron availability by inhibiting cellular iron export. It should be noted that the FS dosage used in mice was lower than that commonly used for treating iron deficiency anemia in humans (1 mg/kg/day in mice versus ~2–3 mg/Kg/day in human or 100–200 mg/day for a 70 kg subject), but still a significant inflammatory response was observed. While the use of FS, and in general of all traditional oral iron formulations, is not an option in the anemia of inflammation, including some conditions characterized by subclinical inflammation like chronic kidney disease [26] or cancer [15], pilot studies has suggested that oral SI is useful in such conditions [17]. Whether the lower propensity to increase hepcidin by SI, as compared to FS, actually represents an advantage for treating human iron deficiency deserves further studies.

In conclusion. we showed that animal models can be used to study the functionality of iron formulations and that SI is efficient and does not cause an inflammatory response compared to the widely used FS. 

## Supplementary Materials

The following are available online at http://www.mdpi.com/2072-6643/10/10/1349/s1. Figure S1: Daily administration of Sucrosomial^®^ Iron and Ferrous Sulfate for 2 weeks did not induce iron accumulation in healthy mice. C57BL/J6 mice (7 weeks old) were treated daily with saline, vehicle, Sucrosomial^®^ Iron and Ferrous Sulfate (1 mg/kg) by gavage for two weeks. Serum iron (A), Transferrin saturation (TSat) (B), Spleen (C) and Liver (D) iron content were analyzed, Figure S2: Sucrosomial^®^ Iron and Ferrous Sulfate were not absorbed by healthy mice after four weeks of daily administration. C57BL/J6 mice (7 weeks old) were treated daily with saline, vehicle, Sucrosomial^®^ Iron and Ferrous Sulfate (1 mg/kg) by gavage for four weeks. Serum iron (A), Transferrin saturation (TSat) (B), Spleen (C) and Liver (D) iron content were analyzed, Figure S3: Fe-Sucrosomial and Fe-Sulfate did not affect the protein level of ferritin and the ferritin iron content both in spleen and liver of healthy mice. Representative image of Western Blotting for Ferritin (top) and representative image of Prussian blue stain plus DAB enhancement of ferritin iron (bottom) in the liver (A) and in the spleen (B) of mice treated with saline, vehicle, Fe-Sucrosomial and Fe-Sulfate. GAPDH and Actin were used as control in liver and spleen respectively, Table S1: Hb and Ht for each mouse during Sucrosomial^®^ Iron and Ferrous Sulfate treatments of healthy mice at the beginning of the treatments (T0) after 2 weeks and after 4 weeks, Table S2: Hb and Ht for each mouse during Sucrosomial^®^ Iron and Ferrous Sulfate treatments of anemic mice at the beginning of the treatments (T0) after 1 week and after 2 weeks.

## References

[1] Kassebaum N.J., Jasrasaria R., Naghavi M., Wulf S.K., Johns N., Lozano R., Regan M., Weatherall D., Chou D.P., Eisele T.P. (2013). A systematic analysis of global anemia burden from 1990 to 2010. Blood.

[2] Tolkien Z., Stecher L., Mander A.P., Pereira D.I., Powell J.J. (2015). Ferrous sulfate supplementation causes significant gastrointestinal side-effects in adults: A systematic review and meta-analysis. PLoS ONE.

[3] Girelli D., Ugolini S., Busti F., Marchi G., Castagna A. (2018). Modern iron replacement therapy: Clinical and pathophysiological insights. Int. J. Hematol..

[4] Toblli J.E., Cao G., Olivieri L., Angerosa M. (2008). Comparative study of gastrointestinal tract and liver toxicity of ferrous sulfate, iron amino chelate and iron polymaltose complex in normal rats. Pharmacology.

[5] Gulec S., Anderson G.J., Collins J.F. (2014). Mechanistic and regulatory aspects of intestinal iron absorption. Am. J. Physiol. Gastrointest. Liver Physiol..

[6] Zimmermann M.B., Chassard C., Rohner F., N’goran E.K., Nindjin C., Dostal A., Utzinger J., Ghattas H., Lacroix C., Hurrell R.F. (2010). The effects of iron fortification on the gut microbiota in african children: A randomized controlled trial in cote d’ivoire. Am. J. Clin. Nutr..

[7] Jaeggi T., Kortman G.A., Moretti D., Chassard C., Holding P., Dostal A., Boekhorst J., Timmerman H.M., Swinkels D.W., Tjalsma H. (2014). Iron fortification adversely affects the gut microbiome, increases pathogen abundance and induces intestinal inflammation in kenyan infants. Gut.

[8] Lane D.J., Merlot A.M., Huang M.L., Bae D.H., Jansson P.J., Sahni S., Kalinowski D.S., Richardson D.R. (2015). Cellular iron uptake, trafficking and metabolism: Key molecules and mechanisms and their roles in disease. Biochim. Biophys. Acta (BBA) Mol. Cell Res..

[9] Lynch S. (2007). Influence of infection/inflammation, thalassemia and nutritional status on iron absorption. Int. J. Vitam. Nutr. Res..

[10] Weiss G., Gordeuk V.R. (2005). Benefits and risks of iron therapy for chronic anaemias. Eur. J. Clin. Invest..

[11] Fabiano A., Brilli E., Fogli S., Beconcini D., Carpi S., Tarantino G., Zambito Y. (2018). Sucrosomial^®^ iron absorption studied by in vitro and ex-vivo models. Eur. J. Pharm. Sci..

[12] Elli L., Ferretti F., Branchi F., Tomba C., Lombardo V., Scricciolo A., Doneda L., Roncoroni L. (2018). Sucrosomial iron supplementation in anemic patients with celiac disease not tolerating oral ferrous sulfate: A prospective study. Nutrients.

[13] Ciudin A., Simó-Servat O., Balibrea J.M., Vilallonga R., Hernandez C., Simó R., Mesa J. (2018). Response to oral sucrosomial iron supplementation in patients undergoing bariatric surgery. The bari-fer study. Endocrinol. Diabetes Nutr..

[14] Giordano G., Mondello P., Tambaro R., Perrotta N., D’Amico F., D’Aveta A., Berardi G., Carabellese B., Patriarca A., Corbi G.M. (2015). Biosimilar epoetin α is as effective as originator epoetin-α plus liposomal iron (sideral^®^), vitamin b12 and folates in patients with refractory anemia: A retrospective real-life approach. Mol. Clin. Oncol..

[15] Barni S., Gascòn P., Petrelli F., García-Erce J.A., Pedrazzoli P., Rosti G., Giordano G., Mafodda A., Múñoz M. (2017). Position paper on management of iron deficiency in adult cancer patients. Expert Rev. Hematol..

[16] Mafodda A., Giuffrida D., Prestifilippo A., Azzarello D., Giannicola R., Mare M., Maisano R. (2017). Oral sucrosomial iron versus intravenous iron in anemic cancer patients without iron deficiency receiving darbepoetin alfa: A pilot study. Support. Care Cancer.

[17] Pisani A., Riccio E., Sabbatini M., Andreucci M., Del Rio A., Visciano B. (2015). Effect of oral liposomal iron versus intravenous iron for treatment of iron deficiency anaemia in ckd patients: A randomized trial. Nephrol. Dial. Transplant..

[18] Capra A.P., Ferro E., Cannavo L., La Rosa M.A., Zirilli G. (2017). A child with severe iron-deficiency anemia and a complex tmprss6 genotype. Hematology.

[19] Castagna A., Campostrini N., Zaninotto F., Girelli D. (2010). Hepcidin assay in serum by seldi-tof-ms and other approaches. J. Proteom..

[20] Poli M., Asperti M., Naggi A., Campostrini N., Girelli D., Corbella M., Benzi M., Besson-Fournier C., Coppin H., Maccarinelli F. (2014). Glycol-split nonanticoagulant heparins are inhibitors of hepcidin expression in vitro and in vivo. Blood.

[21] Girelli D., Nemeth E., Swinkels D.W. (2016). Hepcidin in the diagnosis of iron disorders. Blood.

[22] Udali S., Castagna A., Corbella M., Ruzzenente A., Moruzzi S., Mazzi F., Campagnaro T., Santis D., Franceschi A., Pattini P. (2018). Hepcidin and dna promoter methylation in hepatocellular carcinoma. Eur. J. Clin. Investig..

[23] Roetto A., Di Cunto F., Pellegrino R., Hirsch E., Azzolino O., Bondi A., Defilippi I., Carturan S., Miniscalco B., Riondato F. (2010). Comparison of 3 tfr2-deficient murine models suggests distinct functions for tfr2-alpha and tfr2-beta isoforms in different tissues. Blood.

[24] Hirsh M., Konijn A.M., Iancu T.C. (2002). Acquisition, storage and release of iron by cultured human hepatoma cells. J. Hepatol..

[25] Nemeth E., Ganz T. (2006). Regulation of iron metabolism by hepcidin. Annu. Rev. Nutr..

[26] Ketteler M., Block G.A., Evenepoel P., Fukagawa M., Herzog C.A., McCann L., Moe S.M., Shroff R., Tonelli M.A., Toussaint N.D. (2017). Executive summary of the 2017 kdigo chronic kidney disease-mineral and bone disorder (CKD-MBD) guideline update: What’s changed and why it matters. Kidney Int..

