# Supplementary material for: Sucrosomial® Iron Supplementation in Mice: Effects on Blood Parameters, Hepcidin, and Inflammation
Source: Nutrients. 2018 Sep 21;10(10):1349. doi: 10.3390/nu10101349 (PMC6213119; doi:10.3390/nu10101349)
Supplement: Supplementary file 1 [file nutrients-10-01349-s001.pdf]

Supplemental Material

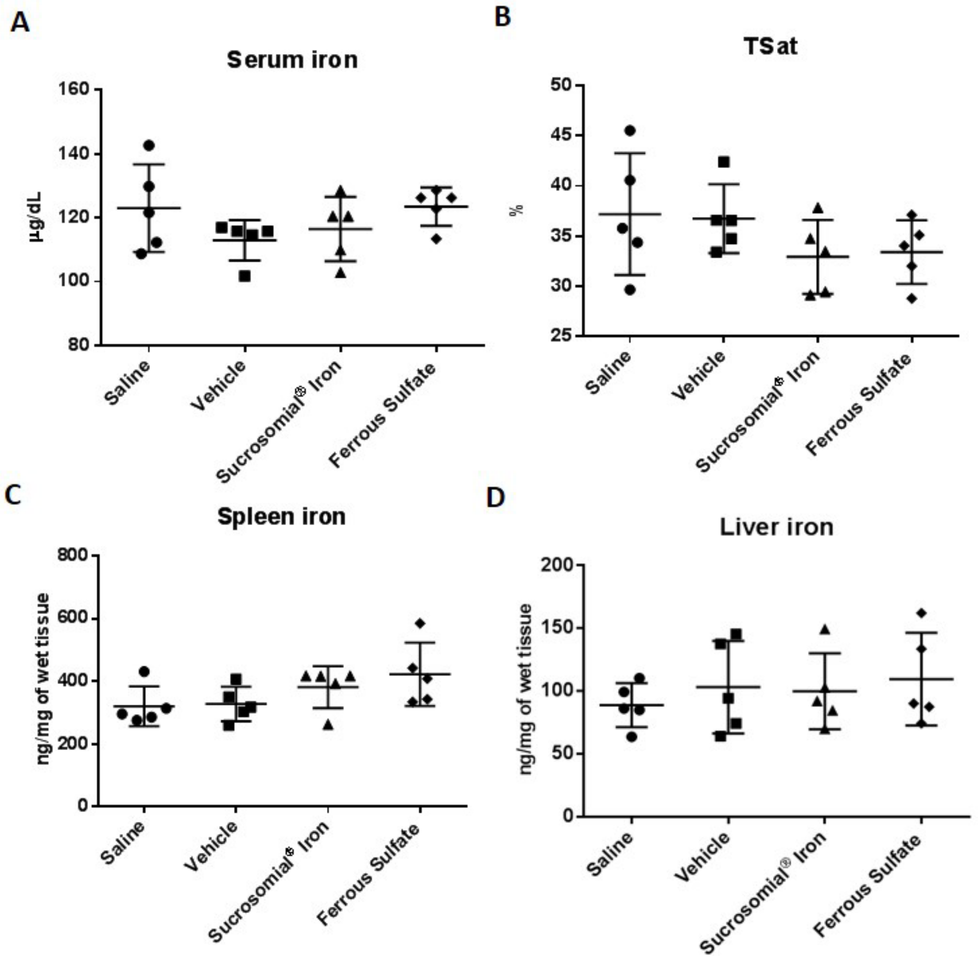

Figure S1: Daily administration of Sucrosomial® Iron and Ferrous Sulfate for 2 weeks did not induce iron accumulation in healthy mice. C57BL/J6 mice (7 weeks old) were treated daily with saline, vehicle, Sucrosomial® Iron and Ferrous Sulfate (1 mg/kg) by gavage for two weeks. Serum iron (A), Transferrin saturation (TSat) (B), Spleen (C) and Liver (D) iron content were analyzed.

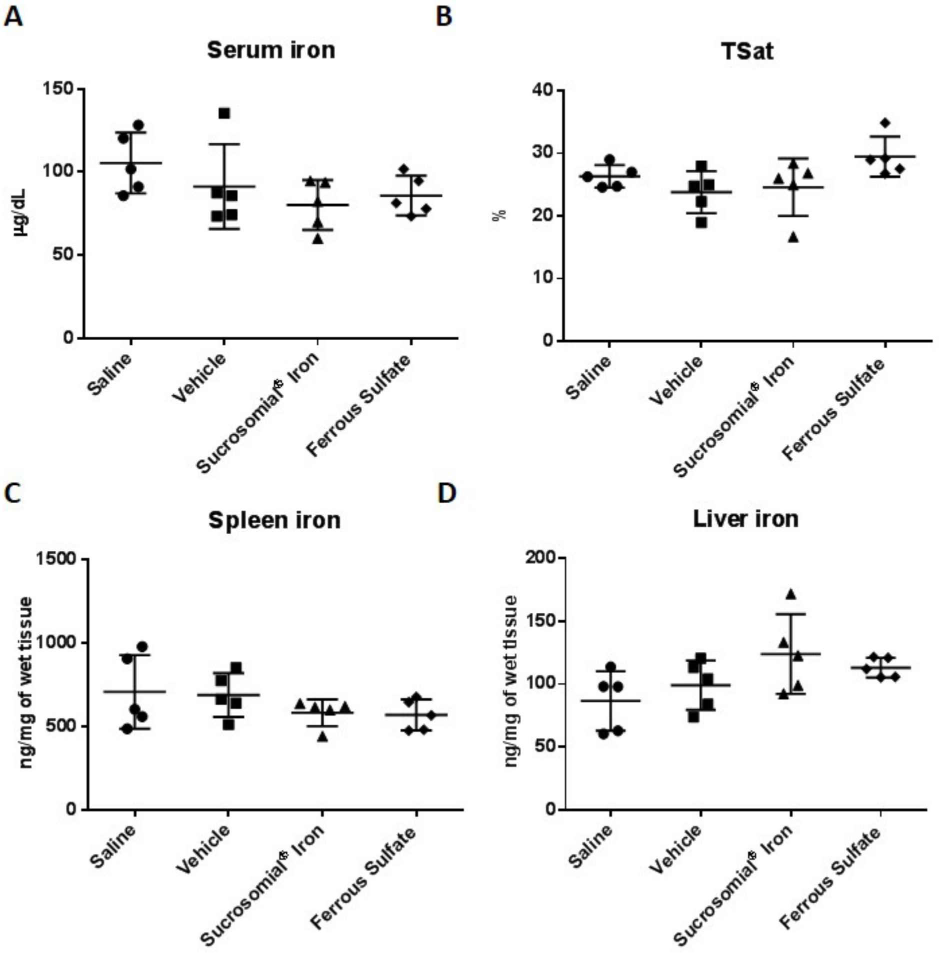

Figure S2: Sucrosomial® Iron and Ferrous Sulfate were not absorbed by healthy mice after four weeks of daily administration. C57BL/J6 mice (7 weeks old) were treated daily with saline, vehicle, Sucrosomial® Iron and Ferrous Sulfate (1mg/kg) by gavage for four weeks. Serum iron (A), Transferrin saturation (TSat) (B), Spleen (C) and Liver (D) iron content were analyzed.

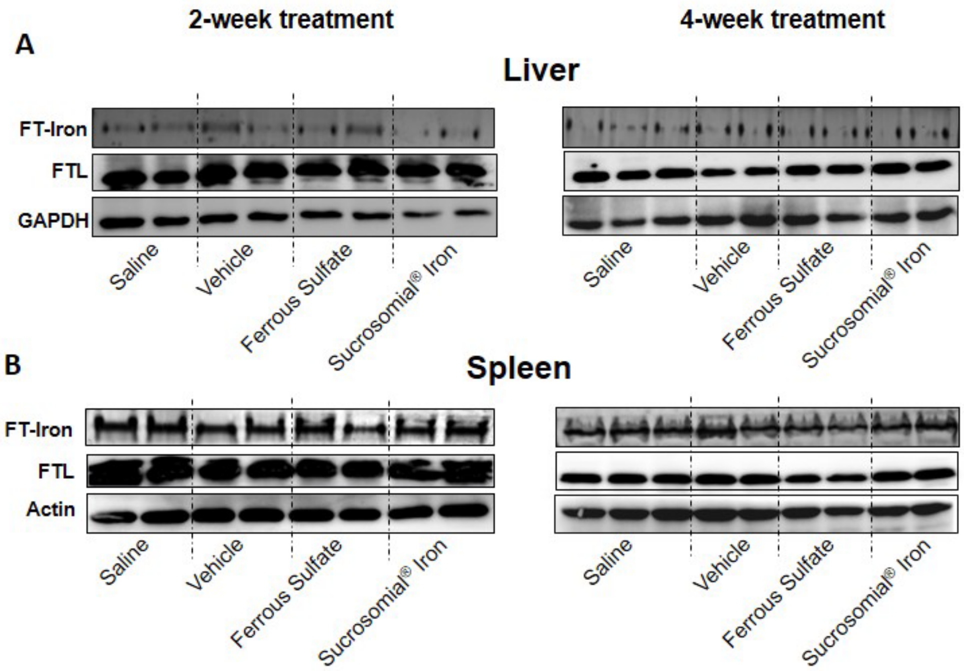

Figure S3: Fe-Sucrosomial and Fe-Sulfate did not affect the protein level of ferritin and the ferritin iron content both in spleen and liver of healthy mice. Representative image of Western Blotting for Ferritin (top) and representative image of Prussian blue stain plus DAB enhancement of ferritin iron (bottom) in the liver (A) and in the spleen (B) of mice treated with saline, vehicle, Fe-Sucrosomial and Fe-Sulfate. GAPDH and Actin were used as control in liver and spleen respectively.

	T0		2 weeks		4 weeks	
	Hb g/dL	Ht	Hb g/dL	Ht	Hb g/dL	Ht
Saline	15,8	46%	15,7	43%	15,7	46%
	14,9	43%	14,8	43%	15,1	44%
	15,5	46%	15	45%	15,6	46%
	16,2	48%	16,1	48%	16,5	48%
	17	50%	17	50%	16,9	49%
Vehicle	16,5	48%	16,5	48%	16,7	48%
	17,4	51%	17,3	51%	16,9	49%
	16,4	47%	16,2	46%	16,4	48%
	16,8	48%	16,6	48%	16,8	49%
	15,7	46%	15,5	46%	15,8	48%
SI	16,2	48%	16,1	47%	15,9	47%
	16,1	47%	15,2	45%	16,2	47%
	15,2	45%	14,3	42%	16	47%
	17	50%	16,9	49%	16,8	49%
	16,8	49%	16,9	49%	17	50%
FS	17	50%	15,4	45%	17	50%
	16,9	50%	15,5	46%	15,1	44%
	16,7	49%	15,6	46%	15,3	45%
	15,5	46%	15,4	46%	15,5	46%
	16,2	48%	16	47%	16,3	48%

SI = Sucrosomial® Iron; FS = Ferrous Sulfate

Table S1: Hb and Ht for each mouse during Sucrosomial® Iron and Ferrous Sulfate treatments of healthy mice at the beginning of the treatments (T0) after 2 weeks and after 4 weeks.

	T0		1 week		2 weeks	
	Hb g/dL	Ht	Hb g/dL	Ht	Hb g/dL	Ht
Saline	11,3	33%	11,5	33%	11,7	33%
	12,1	35%	12,5	37%	12,6	37%
	12	35%	12,2	36%	12,3	36%
	12,5	37%	12,2	36%	12	35%
	12,6	37%	13	38%	13,4	39%
	11,8	33%	11,8	33%	11,8	33%
	9,6	28%	10	29%	10,1	29%
	11,3	33%	11,5	33%	11,7	33%
Vehicle	12,3	36%	12,8	38%	13,3	39%
	12,7	37%	12	35%	11,3	33%
	8,6	26%	9	26%	9,2	27%
	12,4	36%	12,8	38%	13,4	39%
	11,8	33%	12,1	35%	12,6	38%
	10,3	30%	10,5	31%	11,3	33%
	11,5	33%	11,5	33%	11,4	33%
	11,3	33%	11	32%	11,5	33%
SI	9,9	29%	11,6	34%	14,5	43%
	12,1	36%	14,9	44%	16,5	49%
	8,7	26%	11,5	34%	14,9	44%
	8,2	24%	10,7	32%	14,9	44%
	10,5	31%	12,7	38%	16,6	49%
	12,3	36%	14,8	43%	16,9	49%
	11,1	32%	13,5	39%	15,6	46%
	11,7	33%	14	42%	16,5	48%
FS	8,2	24%	12,6	37%	14,7	43%
	9	26%	12,4	36%	14,4	42%
	10,3	30%	14,4	42%	16,3	48%
	12,1	36%	14,9	44%	16,5	48%
	12,7	37%	15,7	46%	16,8	49%
	11,2	32%	15,2	44%	16,9	49%
	12,3	36%	14,3	42%	16,2	47%
	12,1	36%	14,8	43%	16,4	48%

SI = Sucrosomial® Iron; FS = Ferrous Sulfate

Table S2: Hb and Ht for each mouse during Sucrosomial® Iron and Ferrous Sulfate treatments of anemic mice at the beginning of the treatments (T0) after 1 week and after 2 weeks.
